# Adhesive Bacteria in the Terminal Ileum of Children Correlates With Increasing Th17 Cell Activation

**DOI:** 10.3389/fphar.2020.588560

**Published:** 2020-11-30

**Authors:** Bo Chen, Diya Ye, Lingling Luo, Weirong Liu, Kerong Peng, Xiaoli Shu, Weizhong Gu, Xiaojun Wang, Charlie Xiang, Mizu Jiang

**Affiliations:** ^1^ Gastrointestinal Lab, Children’s Hospital, Zhejiang University School of Medicine, National Clinical Research Center for Child, National Children's Regional Medical Center, Hangzhou, China; ^2^ Shaoxing People’s Hospital, Shaoxing, China; ^3^ Collaborative Innovation Center for Diagnosis and Treatment of Infectious Diseases, State Key Laboratory for Diagnosis and Treatment of Infectious Diseases, The First Affiliated Hospital, School of Medicine, Zhejiang University, Hangzhou, China

**Keywords:** Th17 cells, adhesive bacteria, terminal ileum, Crohn’s disease, SIgA

## Abstract

Humans and symbiotic bacteria are interdependent and co-evolved for millions of years. These bacteria communicate with human hosts in the gut in a contact-independent metabolite. Because most intestinal bacteria are non-adhesive, they do not penetrate the mucus layer and are not directly in contact with epithelial cells (ECs). Here, we found that there are adhesive bacteria attached to the Children's terminal ileum. And we compared the immune factors of non-adhesive bacteria in the children ileum with adhesive bacteria as well. Stimulated Th17 cell associated with adherent bacteria in the ileum ECs. SIgA responses are similar to those roles in mouse experiments. Immunohistochemical analysis confirmed that the expression of *SAA1, IL-2, IL-17A, foxp3, RORγt, TGFβ*, and protein increased in Th17 cells. Finally, we used 16S rRNA genes 454 pyrosequencing to analyze the differences in bacterial communities between adhesive and non-adhesive bacteria in the ileum. Ileum with adherent bacteria demonstrated increased mucosa-related bacteria, such as *Clostridium, Ruminococcus, Veillonella, Butyricimonas*, and *Prevotella*. We believe that adhesive bacteria in children’s terminal ileum associated with an increased Th17 cell activation and luminal secretory IgA. Adhesive bacteria very closely adhere to terminal ileum of children. They may play important role in human gut immunity and Crohn’s disease.

## Introduction

Trillions of bacteria are present in our bodies (Sender et al., 2016). These bacteria genomes encode a number of genes not naturally existing in the host, regulate host gut gene expression and affect the differentiation and maturation of the host immune system (Mazmanian et al., 2005). The intestinal walls in humans are joined between the luminal contents and the epithelium, and the mucus layer is separated from the gut bacteria in the intestinal epithelial cells (Meddings, 2008). Past research has found that penetrating bacteria in the intestinal mucosa may cause diseases in humans, such as ulcerative colitis (van der Waaij et al., 2005; Caselli et al., 2013; Johansson et al., 2014). The adhesion features of intestinal bacteria are key factors for inducing Th17 cells (Atarashi et al., 2015). One individual-based model demonstrated that hosts select specific bacteria using adhesion (McLoughlin et al., 2016), because adhesion provides a competitive advantage within host-associated communities (Schluter et al., 2015). However, whether bacteria in intestinal mucosal tissue are both penetrative and adhesive, as well as the effect human gut immunity like SFB induce Th17 cells in mice (Gaboriau-Routhiau et al., 2009; Ivanov et al., 2009), is unknown.

Segmented filamentous bacteria (SFB), or *Candidatus Savagella* (Fernández et al., 2013) in mice are both penetrative and adhesive. A large number of SFB penetrated the villi of wild-type mice during weaning, and induced Th17 cell differentiation and secretory immunoglobulin (SIgA) secretion (Ivanov et al., 2009). SFB enhanced the maturity of the immune system and regulated the immune balance in mice. However, to our knowledge, no study has yet tried to identify that SFB and other bacteria penetrate or adhere to the human ileum. To better understand this issue, we used Scanning Electron Microscopy (SEM) images and observed adhesive bacteria and other mucosa-associated bacteria that penetrate and adhere to the ileum in human. We hypothesized that these bacteria play an important role in human gut immunity, are similar to that of SFB and other adhesive bacteria in mice. We examined Th17 cells associated cytokines *SAA1, IL-17, foxp3, TGFβ, IL-22, RORγt* and host B cells associated cytokine SIgA. To understand the main roles of the mucosa-associated bacteria, we identified the microbiota flora structure non-adhesive and adhesive bacteria of terminal ileum by using 16S rDNA 454 pyrosequencing.

## Materials and Methods

### Subjects

We profiled 106 specimens from the terminal ileum in patients ranging from 6 to 180 months of age by colonic endoscopy because of digestive symptoms such as diarrhea, hematochezia, and abdominal pain. Exclusion criteria: antibiotics used in the last 2 weeks. Recruitment was conducted in the clinic under the protocol approved by the Ethics Committee of Children's Hospital of Zhejiang University School of Medicine. Written informed consent was provided by the parents and from the children as appropriate.

### Scanning Electron Microscopy

The terminal ileum biopsies obtained from endoscopy was directly fixed in 4% glutaraldehyde buffer, sample preparation process is described in the documentation (Brandi et al., 1996; Yu et al., 2013). After coating the gold-palladium film (Chen et al., 2018) on the sample, observe the sample at 20 kV under the H-9500 Hitachi SEM microscope.

### DNA Extraction 16s rRNA Gene 454 Pyrosequencing

QIAamp DNA Stool mini kit (Qiagen, Germany) was used for DNA extractions according to the instructions, and then PCR amplified with bacterial genomic DNA. NanoDrop ND-2000 (NanoDrop Products, United States) was used to quantify DNA. For each DNA sample, the 16S rRNA gene was amplified using a fusion primer set specific for V3–V5 hypervariable regions (F: 5-′TCC​TAC​GGG​AGG​CAG​CAG-3′ and R: 5′-TGT​GCG​GGC​CCC​CGT​CAA​TT-3′) and contained adaptors, key sequences and barcode (Multiple Identifier) sequences as described by the 454 Sequencing System Guidelines for Amplicon Experimental Design (Roche), according to the following protocol: 5 min at 94°C, 27 cycles of 30 s at 94°C, 45 s at 55°C and 1 min at 72°C, followed by a final extension of 7 min at 72°C. The 454 pyrosequencing was determined using the GS FLX+ system and the XL+ chemistry following the manufacturer’s recommendations (Roche 454). Further processing was conducted in a data curation pipeline implemented in QIIME 1.7.0 as pick_closed_reference_otus.py (Caporaso et al., 2010). In summary, this pipeline chose OTUs using a reference-based method and constructs from an OTU table. Taxonomy was assigned using the Green genes predefined taxonomy map of the reference sequence OTUs to taxonomy (McDonald et al., 2012; Vazquez-Baeza et al., 2013). The resulting OTU tables were checked for mislabeling (Knights et al., 2011) and contamination, and further microbial community analysis and principal coordinates beta diversity visualizations were created using Emperor (Knights et al., 2011). A mean sequence depth of 19,914 sequences per sample was obtained, and samples with fewer than 3,000 filtered sequences were excluded from analysis. Alpha- and beta-diversity were calculated using QIIME 1.6.0 (Caporaso et al., 2010), and PcoA plots were produced using Emperor (http://qiime.org/emperor).

### Immunohistochemistry

Biopsy specimens were cut at 5 μm and fixed in 4% paraformaldehyde overnight. For the staining process, tissues were cryoprotected with 30% sucrose in PBS overnight. Two unacquainted pathologists, Weizhong Gu and Xiaojun Wang, who are blinded to score evaluated the results of immunohistochemical staining. Only nuclear staining was considered positive. The scoring rules are as follows: 0 (no detectable staining); 1 (25% positive cells); 2 (25–49% positive cells); 3 (50–74% positive cells); and 4 (75% positive cells). The densities of IL-22, IL-17, foxp3, TGFβ, IL-22, and RORγt positive cells in the surface epithelium and lamina propria were determined by numbers of stained cells per mm^2^ of lamina propria. All data were expressed as mean ± SD (standard deviation) using SPSS 20.0.

### Enzyme Linked Immunosorbent Assay

Measure the total concentration of the fluid in the SIgA chamber, centrifuge approximately 500 μl of sample for 1,000 min at 1,000 × g at 4°C. The sample was diluted 1/10 and serially diluted two times. Human immunoglobulin IgA ELISA kit (Elabscience, China) was used to quantify total SIgA according to the manufacturer's instructions (Chen et al., 2018).

### RNA Extraction and Gene Expression Analysis

Total RNA was extracted from Biopsy specimens of the terminal ileum using Trizol reagent (Life Technologies, United States) and purified using RNeasy mini Kit (Qiagen, Germany).

The amount of gene expression was determined by real-time fluorescent quantitative PCR. RNA was purified from intestinal tissues and qRT-PCR was performed using TaqMan gene expression detection, TaqMan universal PCR master mix (Applied Biosystems) or human-specific primers using SYBR-Green PCR master mix (Applied Biosystems). IL-22 was generated, RORγt IL-17, foxp3 TGFβ, and SAA1 primer sequences were added to the S1 shown in the table. The endogenous control gene is beta actin. Repeated DNA sample assays, each sample normalized to related gene expression and 2-ΔCtβ-actin calculations. The two-tailed *t*-test of unpaired students was statistically significant unless otherwise stated. The *p* value is shown in the graph and the table, and the bar indicates SD (standard deviation). To compare the differences in SIgA levels between adhesive and non-adhesive flora, a non-linear fit curve was introduced into the data using GraphPad Prism 6 (Chen et al., 2018).

## Results

### The Adhesive Bacteria That Penetrate and Adhere to Pediatric Ileum Were Age-Dependent

**FIGURE 1:**
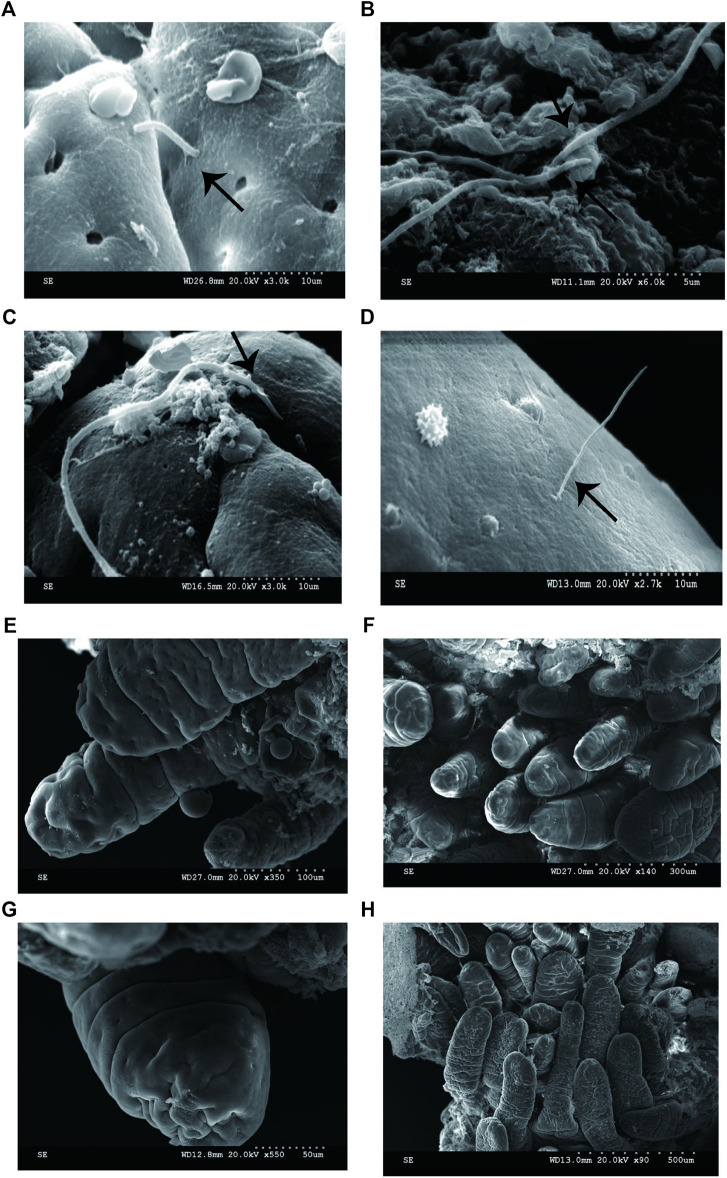
SEM micrographs of human ileal biopsies. The specimens were processed for the observation of microbiota by SEM and as described in Methods. **(A)** SFB-like bacteria inserted and attached tightly to epithelial cell in the terminal ilea by penetrating mucous layer. Sample ID 12,×3000; **(B)** SFB-like bacteria adhered to the ileal mucosa ×6000; **(C)** SFB-like bacteria adhered tightly to the epithelial cell of villi, ×3000; **(D)** SFB-like bacteria superficially penetrated the epithelium of the villi of ilea with both ends outside, ×2700; and **(E--H)** Non-adhesive bacteria observed in the villous epithelium.

**FIGURE 2:**
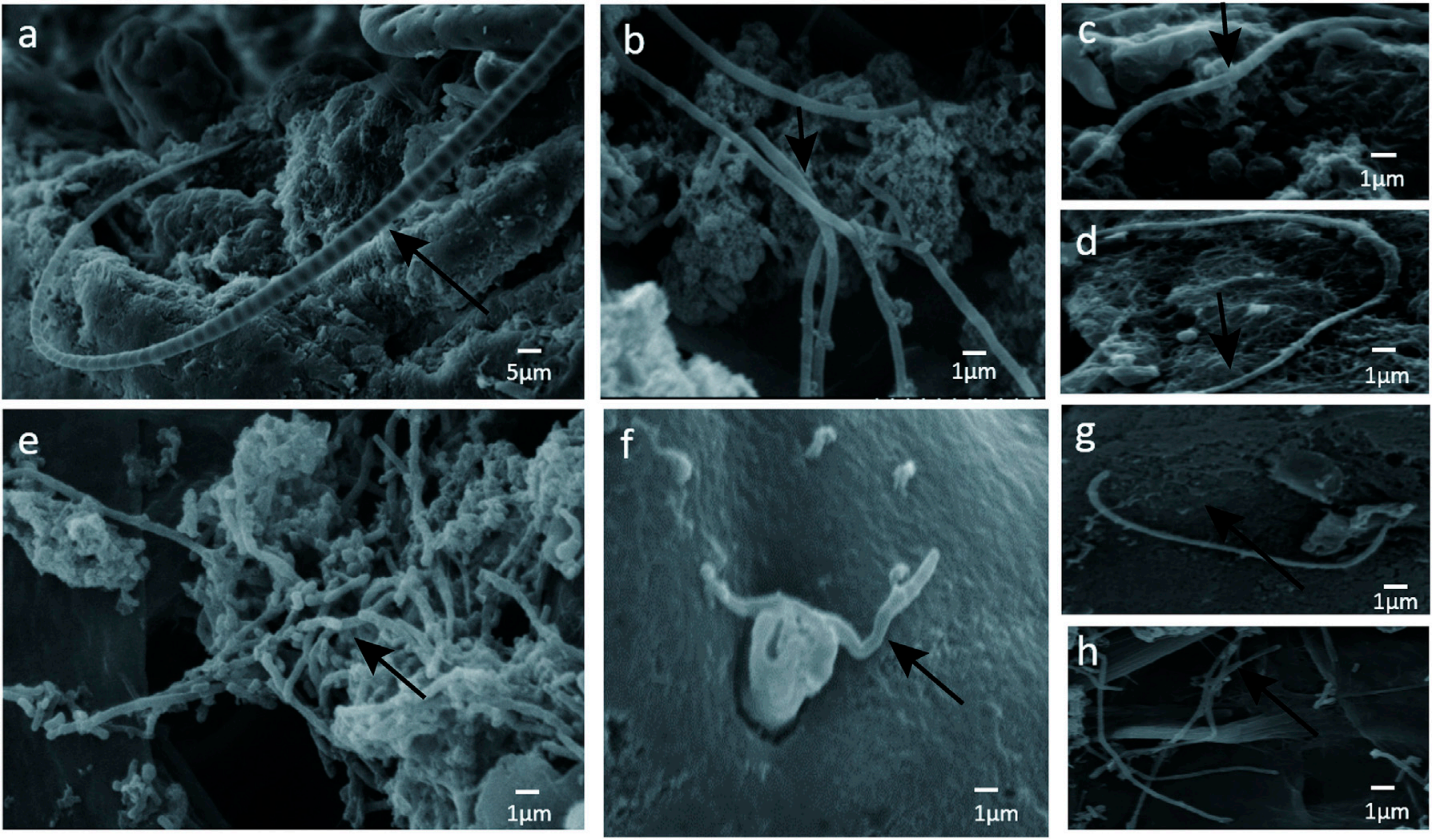
**(A)** SFB-like bacteria attached to epithelial cell in the terminal ilea. Sample ID 12, ×600; **(B --H)** numerous adhesive bacteria observed in the ileal mucosa and epithelial cell ×2500.

To determine if the bacteria penetrated and adhered to small intestinal mucosal tissue, we used SEM images to visualize adhesive bacteria on the surfaces of 14 ileum mucosal biopsy specimens (Figures 1A–D, 2). These bacteria showed that the adhesion in the ileal mucosa was similar to SFB adhesive to ECs in mice. However, most ileum villi were without adhesive bacteria (Figures 1E–H). van der Waaij also did not observe direct contact between bacterial and epithelial cells in adults with normal ileum mucosa (van der Waaij et al., 2005). Among the 14 samples with collected adhesive bacteria, 12 samples were found in the ileum of children 11–36 months. As shown in Table 1, 35.29% of ileum samples collected from children under 11–36 months of age had observable adhesive bacteria on the ileal villi. These results suggested that adhesive and adhesive bacteria penetrated and adhered to 11–36 months pediatric ileum (shortly after weaning).

**TABLE 1:** Age distribution of children with adhesive bacteria on their ileum.

Age (months)	Gender (F/M)	Adhesive bacteria (%)	Adhesive bacteria/total samples
2–10	8/9	5.89	1/17
11–36	16/18	35.29	12/34
37–72	9/10	5.26	1/19
73–108	9/9	0	0/18
109–180	8/10	0	0/18
Total	50/56	13.20	14/106

### The Presence of Adhesive Bacteria Correlated With Induced Th17-Mediated Immune Response Programs in Pediatric Ileum

**FIGURE 3:**
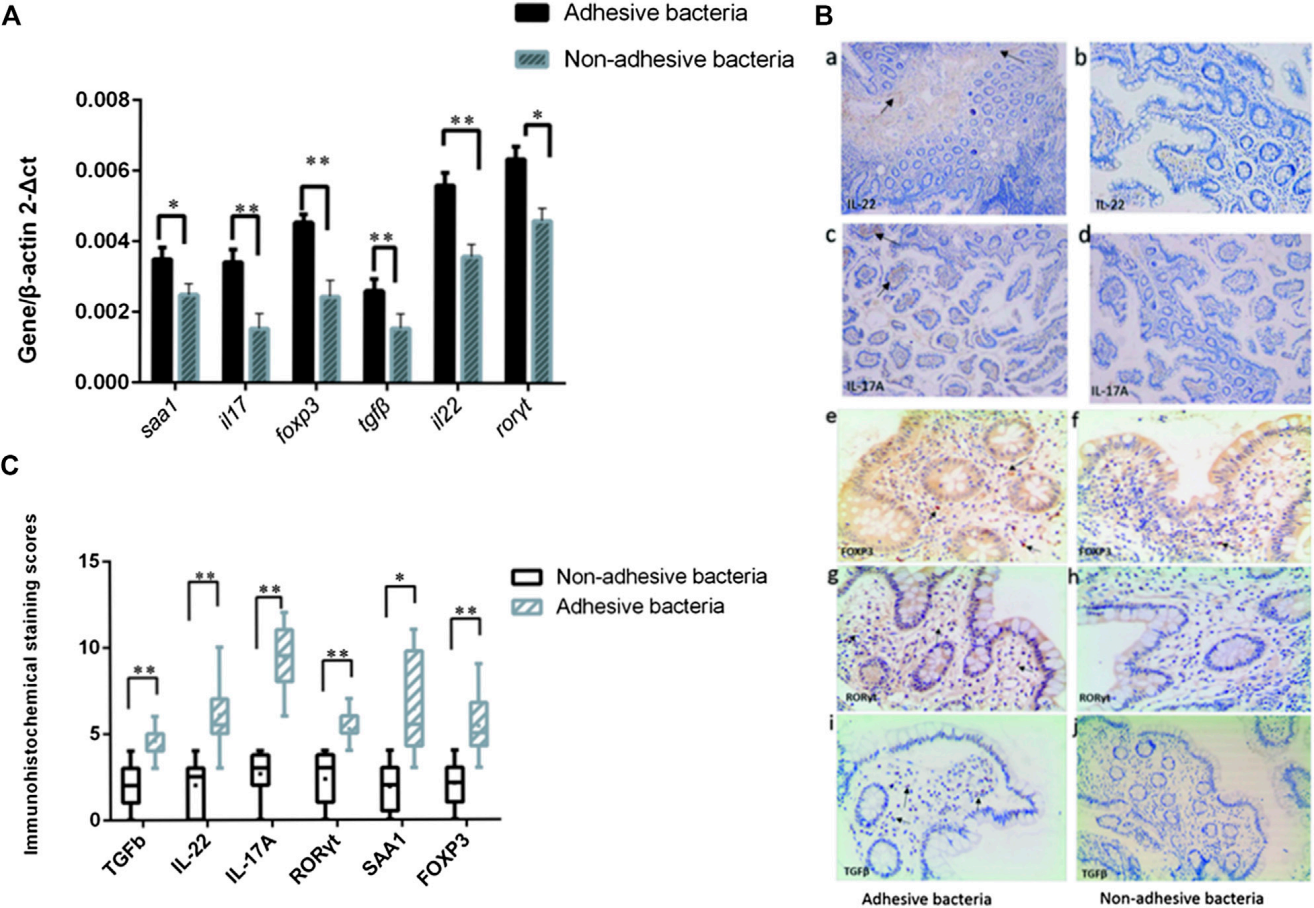
Induction of Th17 cell genes related to the presence of adhesive bacteria. **(A)** qRT-PCR analysis of Th17 cell genes related to the presence of adhesive bacteria. Data are expressed as means (n = 28) and standard deviations. P-values are calculated using the Student's t-test. qRT-PCR was performed as described in the Methods section of this paper. **(B)** **(a--j)** Photographs of the immunohistochemistry in adhesion bacteria and non-adhesion bacteria, IL22, IL17, Foxp3, and RORγt,TGFβ (brown). **(C)** A total score (ranging from 1-25) was obtained by multiplying the staining intensity score (1-5) in the terminal ileum epithelial cells with the positive fraction score (1-5). The densities of IL-22, IL-17, foxp3, TGFβ, IL-22, and RORγt positive cells in the lamina propria and in the surface epithelium were determined by counting the number of stained cells per mm^2^ of lamina propria with adhesive bacteria and non-adhesive bacteria.

Since SFB and adhesive bacteria play key roles in the induction of Th17 cells (Gaboriau-Routhiau et al., 2009; Ivanov et al., 2009). We next examined the influence of adhesive bacteria compared with non-adhesive bacteria in ileum EC immunity gene expression profiles using real-time PCR and immunohistochemical staining (Figure 3). We measured the mRNA *SAA1, IL-17, foxp3, TGFβ, IL-22, RORγt* in 32 of the biopsied samples. qRT-PCR data were expressed as the mean ± SD (*n* = 32). *p* Values were calculated using the Student’s *t*-test. The upregulated transcripts, including the Th17 cell effector cytokines, exhibited increased transcripts SAA1 (a member of the SAA family), interleukin IL-17 and interleukin IL-22 in ileal mucosa with adhesive bacteria (Figure 3). An abundance of SAA1 in adhesive bacteria samples suggested that SAA induction required bacteria adhesion to epithelial cells (ECs). The Th17 cell effector cytokines, such as IL-22 and IL-17, which were required for Th17 cell function, were considered to guard against infections with *Salmonella* and *Citrobacter rodentium* (Atarashi et al., 2015). We next found that the Th17 cell specific transcription regulatory factors, *RORγt* (retinoic acid-related orphan receptor family), Treg specific transcription factors*, F*oxp3 (Fork head box P3), TGFβ (transforming growth factor β) are upregulated in ileum with adhesive bacteria.

These upregulated immunity gene expressions showed that, as in mice, many samples had adhesive microbes that adhered to epithelial cells, and the qRT-PCR of the total ileal tissue exhibited increased amounts of genes participating in T cell differentiation and responses in the adherence bacteria samples.

### The Presence of Adhesive Bacteria Activating SIgA Secretions in the Human Ileum

**FIGURE 4:**
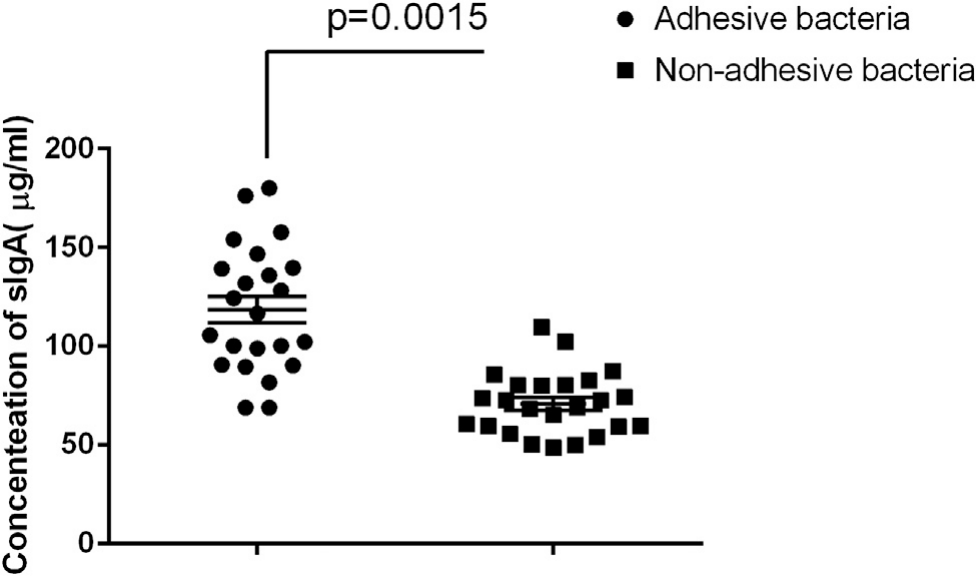
Comparison of SIgA in luminal fluids collected from the adhesive and non-adhesive ileum (n = 12 vs 20). Approximately 500 µl of luminal fluids from each patient were used for the measurement of SIgA as previously described. The supernatants were assayed for total secretory IgA using ELISA with a Human Secretory IgA ELISA Detection Kit as previously described. The SIgA was expressed as µg/ml of fecal materials. Statistical analysis and boxed-plot was performed with the Student's *t-test* using the Graph Pad Prism 6.

Secretory immunoglobulin is an important component of the epithelial barrier because it maintains host intestinal homeostasis (Peterson et al., 2007; Derebe et al., 2014). Many bacteria in the gut are covered by SIgA, and certain adhesive species, such as helicobacter spp. and SFB, are particularly heavily covered by SIgA (Palm et al., 2014). We also quantified SIgA concentrations in intestinal fluids using ELISA age-matched individuals. The results showed that adhesive bacteria ileum fluid had an abundance of SIgA 105.8 μg/ml ± 10.25 μg/ml (mean ± SD, *n* = 12) compared to that of the non-adhesive bacteria ileum fluid 68.53 μg/ml ± 7.24 μg/ml (mean ± SD, *n* = 20) (*p* < 0.001, Figure 4). This indicated that the host secrete more SIgA to maintain intestinal homeostasis.

### The Difference of Microbiota in the Ileum With Adhesive Bacteria and Non-Adhesive Bacteria

**FIGURE 5:**
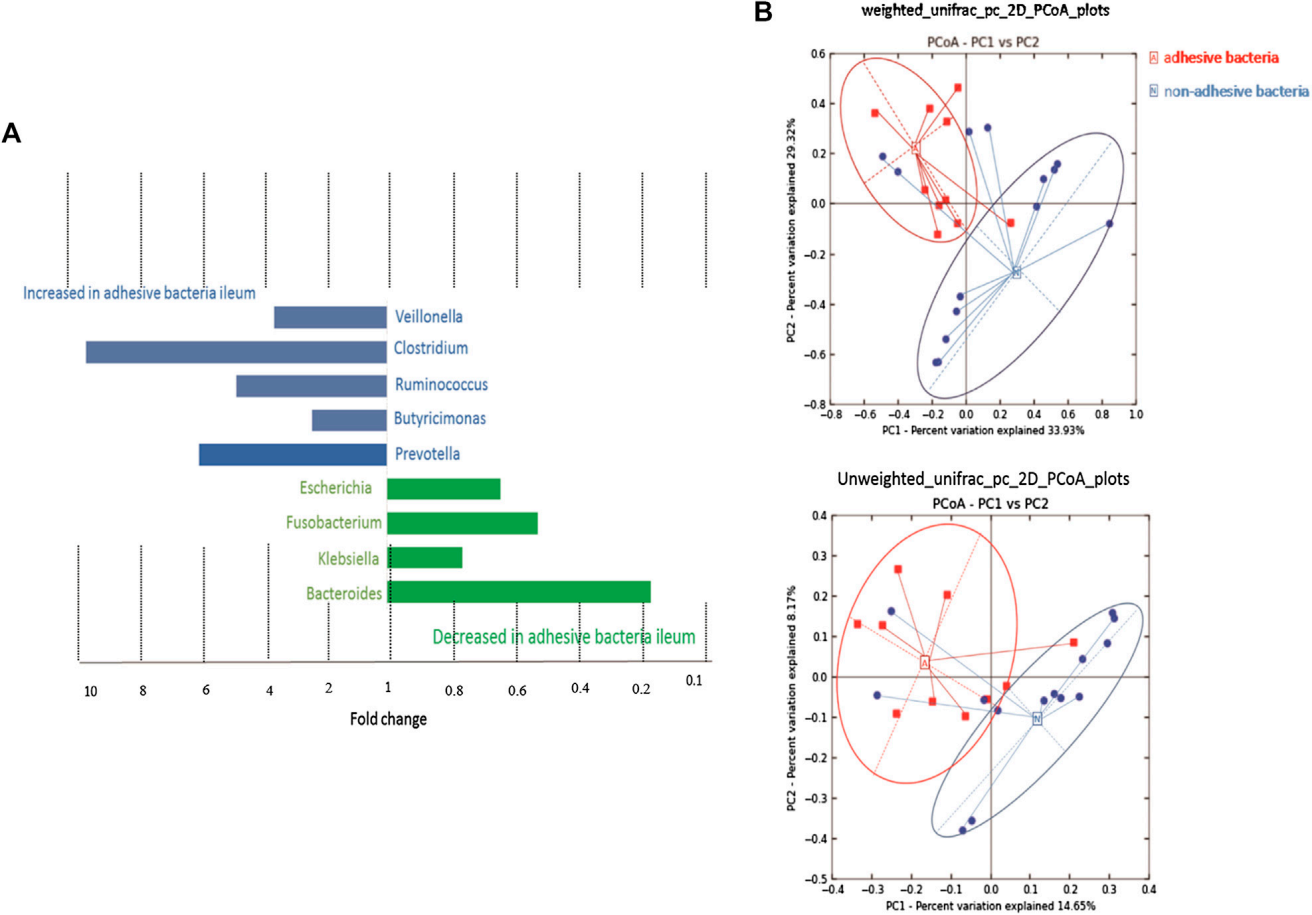
16s rRNA gene 454 pyrosequencing analysis of the gut microbiota. Approximately 500 µg of gut contents from 24 patients were used for the extraction of genomic DNA. Approximately 2 µg of genomic DNA from each patient was used for de novo sequencing and the principal coordinate analysis (PCoA) of the genus profile. The top four genera as the main contributors were determined and plotted by their loadings in these two components. **(A)** Differences in abundance are shown for the taxonomic biomarkers that were detected using a multivariate statistical approach. The fold change for each taxon was calculated by dividing the mean abundance in the cases by that of the controls. Several taxonomic biomarkers measured at both the ileal and the rectal sites were found to be significantly correlated with the adhesive bacteria. **(B)** A set of principal coordinate plots of the unweighted UniFrac distance. PC1, PC2, and PC3 represented the top three principal coordinates that captured most of the diversity, with a fraction of diversity captured by the coordinate, shown as a percent.

A supervised analysis of the 16S rRNA gene sequencing data with LDA effect size compared the bacterial community structure in the ileum with adhesive bacteria to the ileum with non-adhesive bacteria by using an LDA threshold score of 4 (*n* ≥ 5). Adhesive bacteria ileum samples increased the mucosa-associated bacteria *Clostridium, Veillonella, Ruminococcus, Butyricimonas* and *Prevotella,* and decreased *Escherichia, Fusobacterium, Klebsiella, Bacteroides* (Figure 5A). An unweighted UniFrac-based was used to compare the adhesive microbiota and non-adhesive microbiota from pediatric ileum (Figure 5B). The PCoA analysis indicated that the overall diversity in the adhesive microbial composition had greater differences in the samples with non-adhesive microbiota (Table 2). qRT-PCR data were confirmed these findings (Table 3).

**TABLE 2:** Comparison of phylotype coverage and diversity estimation of the 16S rRNA gene libraries at 3% dissimilarity from the pyrosequencing analysis.

Group	Reads	OTUs^a^	Good’s^b^	ACE	95% CI		Chao1	95% CI		Shannon^c^
Non-adhesive bacteria	88,485	2,968	0.978	78,488	76,558.6	77,654.5	52,565.4	50,124.8	55,455.8	3.237
Adhesive bacteria	78,458	2,645	0.972	69,484	67,895.8	68,892.4	48,998.5	48,235.6	49,878.1	2.686

a The operational taxonomic units (OTUs) were defined with 3% dissimilarity level.

b The coverage percentage (Goods), richness estimators (ACE and Chao1) and diversity indices (Shannon) were calculated using Good’s method in the mothur program, respectively.

c The Shannon index of evenness was calculated with formula E = H/ln(S), where H is the Shannon diversity index and S is the total number of sequences in that group.

**TABLE 3:** Primers used in this study.

Gene name	Primer sequence
*FOXP3* F/R	5′-ATC​CGC​CAC​AAC​CTG​AGT​CT-3′/5′-TCC​ACA​CAG​CCC​CCT​TCT​C-3′
*IL-17* F/R	5′-TCC​TAG​GGC​CTG​GCT​TCT​G-3′/5′-AGT​TCG​TTC​TGC​CCC​ATC​AG-3′
*SAA1*	5′-GCT​GAT​CAG​GCT​GCC​AAT​G-3′/5′-GCC​AGC​AGG​TCG​GAA​GTG-3′
*TGFβ*	5′-GCT​GAG​CGC​TTT​TCT​GAT​CCT-3′/5′-CGA​GTG​TGC​TGC AGGTAGACA-3′
*IL-22*	5′-CCC​CAC​TGG​GAC​ACT​TTC​TA-3′/5′-TGG​CCC​TTT​AGG​TAC​TGT​GG-3′
*RORγt*	5′-TGA​GAA​GGA​CAG​GGA​GCC​AA-3′/5′-CCA​CAG​ATT​TTG​CAA​GGG​A3′
*Clostridium*	CloI-F: TACCHRAGGAGGAAGCCAC CloI-R: GTTCTTCCTAATCTCTACGCAT
*Ruminococcus*	F: 5′-AGAGTTTGATCMTGGCTCAG-3′ R: 5′-ACGGCTACCTTGTTACGACTT-3′
*Veillonella*	5′-CAGAAGCAGGTTCCCGTAACTC-3′ 5′-GCCTACCGCAAGTGGCAATA-3′
*Butyricimonas*	Buty1f;5′-GGTGAGTAACACGTGTGCAAC-3′ Buty1r;5′-TACCCCGCCAACTACCTAATG-3′
*Prevotella*	303F: 5′-GAAGGTCCCCCACATTG-3′ 708R: 5′-CAATCGGAGTTCTTCGTG-3′
*Bacteroides*	F: 5′-CGTCCATTAGGCAGTTGGT-3′ R: 5′-CGTAGGAGTTTGGACCGTG-3′
*Klebsiella*	F: 5′-GACGATCCCTAGCTGGTCTG-3′ R: 5′-GTGCAATATTCCCCACTGCT-3′
*E. coli*	F: 5′-AATGATACGGCGACCACCGAGATCT-3′ R: 5′-CAAGCAGAAGACGGCATACGAGAT-3′
*Fusobacterium*	F: 5′CAACCATTACTTTAACTCTACCATGTTCA-3′ R: 5′GTTGACTTTACAGAAGGAGATTATGTAAAAATC-3′

**TABLE 4:** Real time PCR results for human intestinal lavage fluid samples.^a^

Sample	*Bacteroides*	*Klebsiella*	*Clostridium*	*E. coli*	*Prevotella*	*Butyricimonas*	*Veillonella*	*Ruminococcus*	*Fusobacterium*
Adhesive bacteria	12.4^a^	11.64^a^	10.7^a^	11.88^a^	12.09^a^	10.65^a^	11.00^a^	11.22^a^	11.02^a^
Non-adhesive bacteria	13.1^a^	12.03^a^	9.74^a^	12.20^a^	11.28^a^	10.29^a^	10.24^a^	10.56^a^	11.52^a^

a Represents log10 bacteria 16S rRNA gene for each ml of liquid. All data repeated three times.

## Discussion

The ileum epithelial cells and the mucus layer usually separate from the bacteria in the small intestinal. Also, small intestinal bacteria usually cannot penetrate the mucosal layer (van der Waaij et al., 2005), and some bacteria avoid it, such as SFB and pathogenic invasive bacteria. The bacteria had direct contact with the colonic epithelial cells and showed significant associations with ulcerative colitis in humans and mice (Caselli et al., 2013; Johansson et al., 2014). We hypothesized that adhesive and adhesive bacteria also have immunostimulatory and gut immune system maturation roles in humans like that in mice.

### Commensal Bacteria Typically Living in Luminal Fluid and Avoid Direct

Contact with epithelial cells in the human terminal ileum. Atarashi et al. (2015) found that the adhesion of microbes such as *Citrobacter rodentium, Escherichia coli* O157, and 20 bacterial strains from human feces induced Th17 cells in mouse models, are similar to the SFB’s role in the maturation of the host gut immune system (Goto et al., 2014; Lee and Cua, 2014; Schnupf et al., 2015). Based on the individual model which is a hybrid between an individual based model of microbes and a continuum model of solutes (McLoughlin et al., 2016), the results indicated that the host-mediated adhesion increased the competitive advantage of microbes and created a rendezvous for ecological species with slow growth rates (Schnupf et al., 2015). Positive selection through the adhesive can be converted into the negative if the host secreted large amounts of mucus in the matrix. Therefore, the penetration and adhesion bacteria in the human ileum should be studied to understand the impact on the development of the human immune system. Considering that the tight attachment of SFB or other adhesive bacteria, the results showed that host released serum amyloid A (SAA). SAA1 was induced by SFB in the terminal ileum of germ-free (GF) mice. SAA1 induced Th17 cell differentiation in a concentration-dependent manner *in vitro* (Curtiss et al., 2009; Schnupf et al., 2015).

We also investigated whether adhesion was another potential mechanism of the host positive selective. Our findings suggested that microorganisms on the surface of intestinal epithelial cells used adhesion to correlate with immune responses (Schluter et al., 2015).

Our results showed that Th17 cells in the lamina propria were correlated with adhesive microbiota in the human gut. We also observed similar effects in Th17 cells differentiation in mice monocolonized with SFB (Gaboriau-Routhiau et al., 2009; Ivanov et al., 2009). Signals from adhesive bacteria provide a secondary effect, such as the polarization reaction of T helper cells of the Th17 extraction without tissue damage in cases of intestinal immune suppression. Attached SFB stimulated the development of a subset of T helper cells (Faith et al., 2013). Similarly, in that study *Clostridium* also induced regulatory T cells, but the population of many species was more effective than a single isolated or a combination of several species by the reaction of the regulatory T cells. As a result, many other beneficial microbes promoted stable long-term co-existence within the immune system.

Through localized, immune-facilitated and adherence-dependent interactions, the diverse community of microbial symbionts distribution had spatial-temporal heterogeneity in human intestines (Donaldson et al., 2016). The 16S gene rRNA sequencing study of the colonic crypt microbiome demonstrated that the intestinal crypt community included many aerobic bacteria and had a distinct profile relative to the luminal bacteria (Pedron et al., 2012). The human small intestine exhibited lower microbiota diversity than in the colon, and was enriched by certain *Clostridium* spp. members (Palm et al., 2014). In mice, *Proteobacteria* and members of the family *Lactobacillaceae* are enriched in the small intestinal (Lee and Cua, 2014).

Intestinal epithelial cells serve as a physical barrier between microbes and the host's body, and mediate mucous immune responses through the direct perception of microbiota immune responses (Goto et al., 2014), including adaptive immune responses such as SIgA (Peterson et al., 2007; Hapfelmeier et al., 2010), and influence the establishment of the microbiota. The immune system selects appropriate microbiota by innate and adaptive mechanisms, such as SIgA (Caselli et al., 2013). In monoclonization SFB mice model, the numbers of SFB in the terminal ileum changed in an age-dependent manner and was particularly influenced by the IgA concentration in maternal milk during the sucking period and in the luminal content produced by the pups after weaning (Jiang et al., 2001). In this study, we also find the adhesive bacteria increased after weaning. These results show that IgA from maternal milk may regulate the composition of adhesive bacteria in the children ileum.

We propose a model where adhesive bacteria strongly colonize on the small intestines since adhesion permits bacteria to resist displacement by others, and non-adhesive bacteria are more likely to be pushed away from the epithelial cells surface. So long as adhesive bacteria grow on the mucosa layer, the adhesive strain will be dominant. Therefore, adhesion may be a bacterial strategy for colonization in the gut and model (Guzman et al., 1997; Grubb et al., 2009; Nowrouzian et al., 2007).

Few studies addressed the role of immunomodulation by non-pathogens, an aspect that requires these bacteria to have access to the tissue. Based on our observations, we found that the host provided limited space to specific bacteria and that the immune system only allowed adhesive bacteria to access these locations. A particular species close to epithelial cells created a protected microbials despite the rapidly changing conditions in the small intestinal lumen. For example, SFB are members of the symbiotic microbes that penetrate the villi during weaning in wild-type mice.

We showed that adhesive bacteria such as adhesive bacteria in human ileum were special because they were adhesive to the ileal mucosa, are similar to SFB in mice. This behavior resembled that of pathogens and was in contrast with that of most other commensals, which instead remained on the mucus. Adhesive microorganisms have striking characteristic in terms of their morphologies and close proximities to the gut wall. Anchorage into host cells is thought to be necessary for adhesive bacteria to obtain nutrients indispensable for growth but also to induce signals that stimulate the post-natal development of the gut immune system. The findings of these Adhesive and adhesion bacteria in humans are valuable to study their effects on the immune system and human health.

## Conclusion

Adhesive bacteria typically penetrated and adhered to pediatric ileum induced the Th17 cells in the ileum ECs, and triggered SIgA responses. Adhesive bacteria ileum samples exhibited increased amounts of mucosa-associated bacteria, such as *Clostridium, Veillonella, Ruminococcus, Butyricimonas*, and *Prevotella.*

## Data Availability Statement

The datasets of the 16S rRNA gene 454 pyrosequencing were available in the NCBI. The accession number was PRJNA343381.

## Ethics Statement

The studies involving human participants were reviewed and approved by Ethics Committee of Children's Hospital of Zhejiang University School of Medicine. Written informed consent to participate in this study was provided by the participants' legal guardian/next of kin.

## Author Contributions

Study concept, designs and obtained funding (MJ). Drafting of the manuscript, acquisition of data and statistical analysis (BC). Analysis and interpretation of data (BC, LL, DY). Critical revision of the manuscript for important intellectual content (CX, XS). Colonoscopy and clinical data collection (KP, XW, WG).

## Funding

The Scientific Research Fund of National Health and the Family Planning Commission-Major Science and Technology Project of the Zhejiang Province Medical and Health (WKJ-ZJ-1622).

## Conflict of Interest

The authors declare that the research was conducted in the absence of any commercial or financial relationships that could be construed as a potential conflict of interest.
